# Cherubism: An African-Focused Review

**DOI:** 10.3390/children13020295

**Published:** 2026-02-20

**Authors:** Salma Kabbashi, Imaan A. Roomaney, Martin Douglas-Jones, Karen Fieggen, Nakita Laing, Suvarna Indermun, Manogari Chetty

**Affiliations:** 1 Department of Craniofacial Biology, Pathology and Radiology, Faculty of Dentistry, University of the Western Cape, Cape Town 7505, South Africa; skabbashi@uwc.ac.za (S.K.); iroomaney@uwc.ac.za (I.A.R.); mchetty@uwc.ac.za (M.C.); 2 Department of Maxillofacial and Oral Surgery, Faculty of Dentistry, University of the Western Cape, Cape Town 7505, South Africa; mdouglas-jones@uwc.ac.za; 3 Division of Human Genetics, Faculty of Medicine, University of Cape Town, Cape Town 7505, South Africa; karen.fieggen@uct.ac.za (K.F.); n.verkijk@uct.ac.za (N.V.)

**Keywords:** cherubism, Africa, paediatric craniofacial disorders, fibro-osseous lesions, *SH3BP2*, genetic diagnosis, maxillofacial pathology

## Abstract

**Highlights:**

**What are the main findings?**

- The epidemiology of cherubism in Africa cannot be accurately determined from the currently available published data.
- Although symptom onset typically occurs in early childhood, presentation for specialist care is often delayed until adolescence, with significant complications reported.

**What are the implications of the main findings?**

- There is a need for registries and publications on the long term follow up of patients with cherubism to better understand if the natural history of cherubism and whether the phenotype differs from what has been reported in other regions.
- Enhancing genomics literacy, expanding molecular diagnostics and improving multidisciplinary craniofacial care, is needed to improve patient outcomes and ensure African data inform the global understanding of cherubism.

**Abstract:**

Cherubism is a rare fibro-osseous disorder of the jaws that typically presents in early childhood and is recognised as genetically heterogeneous. While the condition is well described in non-African populations, African data and molecular confirmation remain limited. Background/Objectives: This structured narrative review aimed to synthesize published African cases of cherubism by describing patterns of presentation, diagnosis, management, and genetic investigation. Methods: A structured narrative literature review was conducted using PubMed, Scopus, Google Scholar, and African Journals Online. Peer-reviewed case reports and case series describing cherubism in African patients were included. Data extraction followed predefined criteria, capturing demographic features, age at onset and presentation, clinical, radiological and histological findings, management strategies, and the use of molecular genetic testing. Findings were synthesised descriptively. Results: Fourteen studies reporting 20 individual cases from eight African countries were identified, with the majority originating from North Africa. Although symptom onset most commonly occurred in early childhood, the median age at presentation for management was 13.75 years, suggesting delayed access to care. Molecular genetic testing was reported in only two cases, while most diagnoses relied on clinical, radiological, and histopathological features. Surgical intervention was commonly described, with fewer cases managed conservatively. Conclusions: Within the limitations of a structured narrative review based predominantly on published case reports and case series, and constrained by the scarcity of molecularly confirmed cases, the available African literature on cherubism remains limited in scope, geographically skewed, and characterised by incomplete genetic reporting. Recurring features include delayed presentation, reliance on clinical diagnosis, and limited use of molecular testing. These observations reflect gaps in reporting and genetic characterisation rather than population-level patterns, underscoring the need for improved molecular diagnostics, multidisciplinary care, and African registries.

## 1. Introduction

Cherubism (OMIM #118400) is a rare, predominantly autosomal-dominant fibro-osseous dysplasia confined to the mandible and maxilla. It clinically presents in early childhood with painless progressive, bilateral jaw expansion that often stabilizes or regresses after puberty [1,2]. Radiographically, the disease is characterized by bilateral expansile multilocular radiolucencies with cortical thinning, while histology shows a cellular fibrous stroma containing multinucleated giant cells and perivascular collagen cuffing [3]. The entity derives its name from Renaissance art, referring to the characteristic facial appearance of affected individuals, thought to resemble the faces of cherubs—baby angels with plump cheeks and upturned eyes [4].

Cherubism is considered a rare disease worldwide, yet its prevalence remains poorly defined, with most reports originating from case series in Europe, Asia, and North America. Reports from Africa remain limited, despite documented cases across several countries. This apparent scarcity in the African literature may reflect limited case ascertainment and reporting rather than true differences in disease occurrence, although these factors cannot be reliably disentangled. Likely contributing factors include under-recognition at the clinical and community level, diagnostic limitations related to variability in access to specialist and molecular testing, and epidemiologic under-reporting in the published literature [2,5,6]. Establishing an African-focused understanding of the condition is therefore critical, particularly given that craniofacial fibro-osseous lesions such as fibrous dysplasia and giant cell granuloma are more frequently encountered in the African context [7], and may pose significant diagnostic challenges.

## 2. Molecular Genetics and Pathobiology

The disorder is strongly associated with pathogenic variants in the *SH3BP2* gene located on chromosome 4p16.3 [8]. Although penetrance is high, clinical expressivity is variable, ranging from mild or subclinical disease to severe, disfiguring maxillofacial involvement, with male patients generally exhibiting a more pronounced phenotype [9,10].

The canonical genetic basis of cherubism is classically attributed to pathogenic variants of the *SH3BP2* gene within exon 9 (codons Arg415–Gly420). These variants result in a gain-of-function effect, driving enhanced osteoclastogenesis and amplification of downstream inflammatory signalling pathways [10,11]. More recently, the spectrum of genes implicated in cherubism has expanded beyond *SH3BP2* with the identification of biallelic *OGFRL1* loss-of-function variants in autosomal-recessive families, defining a subset of cases not accounted for by *SH3BP2* alterations [12].

In addition, gain-of-function variants in *PLCG2* have been described in *SH3BP2*-negative individuals, with overlapping features of autoinflammatory conditions, thereby broadening the recognised clinical and molecular spectrum of the disease [13].

**Figure 1:**
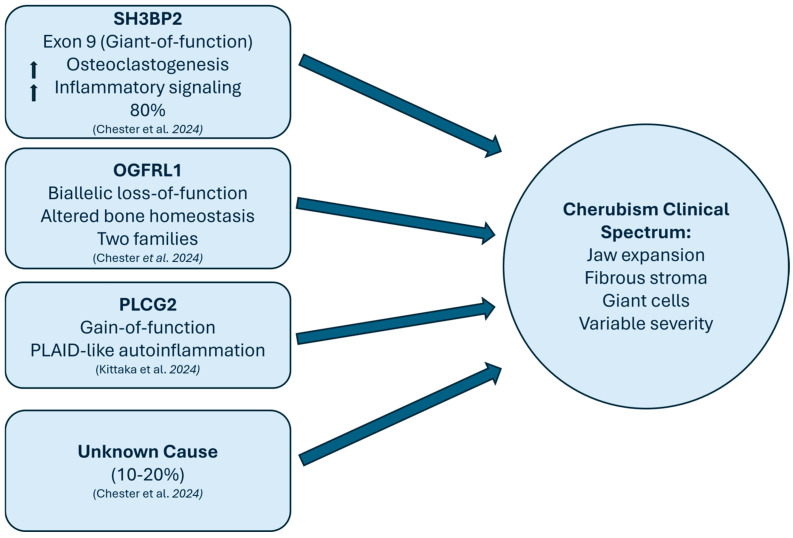
Molecular pathways and genetic heterogeneity in cherubism: Schematic representation of the genetic mechanisms contributing to cherubism’s variable clinical features and highlighting the interplay between bone remodeling and immune signaling [13,14].

Despite these advances, approximately 10–20% of cherubism cases remain without an identified genetic etiology, indicating that the molecular basis of the disorder is not yet fully elucidated [14] (Figure 1). In this context, contemporary diagnostic approaches often incorporate more comprehensive genomic strategies when *SH3BP2* variants are absent when SH3BP2 variants are absent [15].

In African settings, where molecular testing has been reported in only a small number of published cases, the absence of sequencing limits diagnostic certainty and complicates interpretation of observed clinical patterns. Without genetic confirmation, phenotypic variability—such as severity, jaw distribution, or associated features—cannot be reliably linked to underlying molecular mechanisms, and differentiation from histologically overlapping giant cell lesions remains challenging [15].

While much of this molecular knowledge is derived from non-African cohorts, it provides an essential framework for interpreting the limited African data and underscores the importance of comprehensive genetic evaluation in under-studied populations.

Given the global rarity of cherubism and its under-documented presence in Africa, this study was designed as a structured narrative literature review aimed to collate and characterize published African case reports and case series of cherubism. The review was particularly motivated by the growing genetic complexity of cherubism, highlighted by the discovery of variants beyond the canonical *SH3BP2* gene, and the urgent need to contextualize these findings within African populations, where access to molecular diagnostics remains limited.

## 3. Materials and Methods

### 3.1. Study Design and Rationale

This study followed a structured narrative literature review methodology to identify and summarize published reports of cherubism involving patients from African countries. This review sought to (i) determine the extent of published clinical and genetic data on cherubism in African contexts, (ii) identify diagnostic and reporting gaps across the continent, and (iii) highlight priorities for future molecular investigations.

### 3.2. Eligibility Criteria

Inclusion Criteria

Studies were included if they met the following criteria:

- Reported cases of cherubism, diagnosed based on clinical, radiological, histological, or molecular criteria;
- Patients originating from and reported within African countries;
- Consisted of original case reports, case series, or primary studies;
- Were published in peer-reviewed journals;
- Were written in English, French or Arabic languages (English-indexing).

Exclusion Criteria

Studies were excluded if they were:

- Review articles, narrative or systematic, in vitro studies, animal model studies and, reports lacking sufficient patient-level clinical details;
- Editorials, letters, or conference abstracts without full data access.

### 3.3. Literature Sources and Search

A targeted literature search was undertaken using PubMed, Scopus, Google Scholar, and African Journals Online (AJOL) to identify relevant published reports. The Search strategy is included in Supplementary Material Table S1. The search was modified as appropriate for the search syntax of each database. No date restriction was applied. The most recent search was conducted in October 2025. Reference lists of included articles were also screened to identify additional eligible studies. Google Scholar was searched to a depth of 50 pages, with 20 results per page.

### 3.4. Data Collection and Narrative Synthesis

For each included study, the following data were extracted:

- Country and setting;
- Year of publication;
- Study type (case report, series);
- Number of patients;
- Age at onset and diagnosis;
- Clinical features (e.g., jaw expansion, facial swelling);
- Radiological and histological findings;
- Whether molecular testing was performed (yes/no);
- Gene(s) sequenced and variant(s) identified.

Molecular confirmation was defined as the performance of molecular genetic testing in the diagnostic evaluation of cherubism [16]. Genetic testing was considered distinct from pathogenic variant confirmation, as identification of sequence variants requires subsequent independent interpretation and classification to determine clinical significance [16]. Reporting of the specific gene(s) tested and variant(s) identified was recorded separately and was not required for classification as molecularly confirmed.

Reports were cross-checked for overlapping authorship, institutions, patient characteristics, and publication dates to minimise the risk of duplicate case inclusion.

Results were synthesized descriptively using narrative summaries and tabular displays, with a focus on mapping the presence or absence of molecular data across the African continent.

## 4. Results

### 4.1. Study Characteristics

A total of 14 studies were included in this review (Table 1). Two studies were case series, comprising three patients from Algeria [17] and five cases from Egypt [18,19,20,21,22,23]. The remaining 12 studies reported individual cases. The first study was published from Algeria in 1989 [17], while most studies were published within the last decade (*n* = 8) [5,18,19,24,25,26,27,28]. Overall, the included studies reported cases from eight African countries. Egypt contributed the highest number of studies (six studies, 10 cases), followed by Morocco (2 studies, 2 cases), and one study each from Algeria, Democratic Republic of the Congo (DRC), South Africa, Nigeria, Sudan, and Ghana.

Two studies, both originating from North Africa (Egypt and Morocco), reported combined clinical and molecular findings [21,28]. The remaining 18 cases were diagnosed based on clinical, radiographic, and/or histological criteria. All included studies were published in English in peer-reviewed journals.

### 4.2. Patient and Phenotypic Characteristics

Collectively, these studies described 20 individual cases of cherubism. There was a slight male predominance (*n* = 11 males; *n* = 9 females), with ages at presentation ranging from 5 to 24 years. Among the studies reporting age at presentation, the median age at presentation for management was 13.75 years, with 9 cases presenting at or beyond 12 years of age. Age of symptom onset was reported numerically for 17 cases, yielding a median onset age of 4 years; cases with qualitative-only descriptions of onset were excluded from this calculation. Most cases (*n* = 13) reported symptom onset in early childhood (before 5 years of age), with one study noting age of onset shortly after birth [19], and another at 9 months [21]. Only one case reported an age of symptom onset of 12 years [25].

Of the 20 cases, 15 exhibited bimaxillary involvement, four showed isolated mandibular involvement, and one had isolated maxillary involvement [25]. All patients presented with bilateral enlargement. Elfahsi et al. described an asymmetrical onset, with the initial involvement of the left mandible followed by subsequent involvement of the right maxilla [29].

Table 2 provides additional details on family history and clinical findings of the cases. Three studies showed familial types of cherubism with multiple affected family members [17,19,29]. The remaining studies reported no significant family history, with one case noting a reported history of an older sibling who had similar jaw swelling in childhood [29]. Submandibular and cervical lymphadenopathy were reported in five cases [17,19,25]. Biochemical abnormalities, including elevated serum alkaline phosphatase and calcium levels, were documented in three patients [19,20,22]. Ocular involvement was reported in seven cases [16,28] with exophthalmos being the most frequent sign reported. Temtamy et al. described severe ocular symptoms, including bilateral partial optic nerve atrophy, severe vision impairment, bilateral proptosis, conjunctivitis and inability to close the eyes [23]. One study documented a positive history of nasal obstruction [27], while another study reported a patient death from sudden cardiorespiratory arrest with obstructive sleep apnoea proposed as a possible contributing factor [24].

Most of the cases reported in this review underwent surgical management at some stage, including curettage, recontouring, osteotomy, and in one case, maxillectomy [5,17,18,20,23,24,28,29]. Four studies reported conservative monitoring and follow-up [5,19,22,27], with one case eventually requiring surgical intervention due to dental complications [2,5].

## 5. Discussion

This review aimed to synthesise and summarise findings from published African cases of cherubism by describing patterns of presentation, diagnosis, management, and genetic investigation. Only 14 publications were identified, collectively reporting 20 individual cases of cherubism. The majority of cases (14/20; 70%) originated in North Africa, suggesting that cherubism is likely underreported across much of the African continent. This limited and geographically skewed evidence base constrains the generalisability and interpretability of the review’s findings.

### 5.1. Genetically Confirmed Cases: The African Experience

Genetically confirmed cases of cherubism in African populations remain rare. The first molecularly confirmed case was reported in Egypt in 2014, in which a pathogenic *SH3BP2* variant was identified in a five-year-old child [21]. Several years later, another genetically confirmed case was reported in a 12-year-old child from Morocco; however, the specific identified gene was not reported by the authors [28]. Beyond these isolated cases from North Africa, the majority of published African reports have relied on clinical, radiographic, and histological features without molecular confirmation. These include case series or individual reports from Algeria [17], DRC [24], Nigeria [27], Ghana [25], Sudan [26], and South Africa [5], in which diagnoses were based on clinical, histopathological, and radiological criteria, with no molecular testing performed or genetic results reported.

At present, there are no published reports documenting pathogenic variants in *PLCG2* or *OGFRL1* in Africa, both of which have recently been described in non-African cohorts [13,14]. This absence likely reflects under-detection rather than true population differences and highlights a persistent gap between phenotypic recognition and molecular characterization. In resource-constrained settings, a clinical diagnosis of a rare disorder is often considered sufficient to guide management [15]. However, in the presence of atypical phenotypic features, or in regions with a high prevalence of phenotypically similar conditions, molecular confirmation should be considered to refine the diagnosis.

Importantly, the risks and costs associated with invasive histological confirmation may exceed those of targeted molecular testing, particularly given that cherubism-associated lesions are often indistinguishable from other giant cell lesions on histology alone. In clinically ambiguous cases, molecular confirmation may therefore enhance diagnostic clarity, support appropriate counselling, and provide reassurance to both clinicians and patients.

Although the small number of molecularly confirmed cases of cherubism in Africa may partly reflect the overall rarity of the condition, it is more likely attributable to under-reporting and under-characterisation. While the contributing factors can only be inferred, limited genomics literacy among clinicians and uneven access to molecular diagnostic testing represent broader challenges within African craniofacial genetics [24]. Despite the increasing availability of advanced molecular diagnostics in high-income settings, many cases across the continent appear to remain genetically uncharacterised, thereby potentially limiting accurate genetic counselling, cross-population comparisons, and the identification of novel or population-specific variants. Strengthening access to genetic testing in Africa—through collaborative sequencing initiatives, the development of regional genomic reference databases, and the integration of craniofacial genetics into routine clinical practice—would be essential to move beyond purely descriptive reports towards molecularly confirmed diagnoses. Such efforts could not only improve clinical care but also generate critical insights into potential population-specific variation and contribute to a more comprehensive global understanding of the genetic heterogeneity of cherubism and other giant cell lesions.

### 5.2. Phenotypic Characterisation

Clinically, cherubism typically manifests between 2 and 5 years of age, with radiographic changes often detectable even earlier [15]. Although most cases in this review reported symptom onset in early childhood, presentation for clinical management generally occurred much later, with a median age at presentation of 13.75 years; most patients (13/20) sought care only at or after 12 years of age. By contrast, non-African cohorts report clinical presentation as early as 2–3 years of age [30]. This delay may, in part, reflect variability in access to specialist and general dental services in many low- and middle-income settings (LMICs). It may also reflect under-recognition of early, subtle signs by families or healthcare providers, phenotypic variability with less conspicuous early features, or sociocultural influences on health-seeking behaviour [25]. The scarcity of molecular data from African cohorts leaves open the possibility that uncharacterised genetic modifiers or variants may influence disease course, as observed in other craniofacial conditions with African-specific variant spectra [31]. This interpretation is supported by the observation that 10–20% of cases of cherubism worldwide lack an identified causative variant [15].

The proportion of bimaxillary involvement in the included publications was high. Reports from predominantly non-African cohorts have described mandibular-only involvement as the more common pattern [32], with maxillary or bimaxillary expansion generally associated with more extensive phenotypes [2]. In contrast, in the available African cases reviewed here, combined mandibular and maxillary involvement was frequently reported, a pattern that might have an important clinical implications but could also reflect case report publication bias. Maxillary expansion increases the risk of orbital encroachment, and severe ocular complications, such as bilateral partial optic atrophy, marked proptosis, and lagophthalmos, have been documented [19]. Airway compromise has also been described, including a case of sudden cardiorespiratory arrest where obstructive sleep apnoea was suspected [5]. Overall, these findings suggest potential important differences in presentation, although the small number of African cases limits firm conclusions.

### 5.3. Management Considerations

The natural history of cherubism is characterized by the emergence of jaw lesions during early childhood, with progression during periods of active growth [1]. Lesions typically stabilise or regress spontaneously after puberty, often leaving residual facial asymmetry or dental malocclusion [33]. Complications may include tooth displacement, delayed eruption, root resorption, and, in rare severe cases, functional impairment of vision, speech, or breathing due to maxillary involvement [10].

Given the condition’s self-limiting natural history, management of cherubism is generally conservative, with most cases resolving by adolescence. Surgical intervention may typically include contouring or resection and is generally reserved for patients with functional compromise (such as impaired mastication, vision, or speech) or severe aesthetic deformity [2].

Although a few studies described expectant monitoring, most patients in the available literature underwent operative intervention, including osteotomy and, in one instance, maxillectomy, with only four studies adopting a true ‘watch-and-wait’ strategy. These patterns may reflect advanced disease stage or a higher proportion of bimaxillary involvement. Alternatively, it may indicate a lack of publication of less severe cases, as case-based literature tends to preferentially report more complex or surgically managed presentations. Accordingly, management trends derived from the case reports included in this review may not be fully representative of standard practice or population-level decision-making.

Pharmacological approaches remain experimental, with only small case series assessing agents such as calcitonin, interferon-α, or bisphosphonates. To date, the evidence supporting pharmacological management remains inconclusive [8]. No pharmacological interventions have been reported in African patients, where management has largely been described as centered on surgery and supportive care.

Importantly, African studies have suggested that diagnostic challenges including misdiagnosis, or confusion with other giant cell-rich jaw lesions (such as central giant cell granuloma or fibrous dysplasia) may delay appropriate surveillance or management, emphasising the need for increased awareness and diagnostic accuracy [25]. This reflects a broader theme: while the natural history of cherubism is universal, the context of care in Africa, including variable access to molecular testing and constrained surgical infrastructure, warrants exploration.

It should be noted that interpretation of these observations requires caution, in light of the limited and heterogeneous nature of the available literature. Additional limitations include publication bias inherent to case-based reports, small sample size, and limited molecular confirmation. Furthermore, the literature search was conducted using English-language indexed terms only, which may have contributed to under-ascertainment of relevant African reports published exclusively in French or Arabic.

### 5.4. Future Directions for Africa

Improving rare disease literacy and capacity in rare disease research and management are vital first steps toward understanding and tackling rare craniofacial conditions, including cherubism. Establishing African registries, biobanks, and longitudinal follow-up studies would address current gaps in reporting and under-sequencing, support clinical trials, and provide the foundation for functional genomic research, particularly when linked to broader genomic initiatives and continental platforms such as H3Africa [34].

Future progress in cherubism care will rely on aligning global advances in molecular genetics with the needs and realities of African clinical settings. Globally, there is an increasing shift toward precision diagnosis and targeted management, emphasising the growing importance of early molecular confirmation [35]. The limited molecular testing evident in this review highlights the need to expand access to genetic testing, particularly as sequencing costs continue to fall relative to the financial and clinical burden of surgical intervention and its potential sequelae. Targeted testing for *SH3BP2*, *OGFRL1*, and *PLCG2* in affected patients and families, demonstrates how molecular diagnostics can be directly integrated into clinical care (Figure 1).

Where molecular studies are available, a tiered genomics strategy offers a pragmatic pathway: targeted sequencing of *SH3BP2* exon 9 as an initial step in resource-limited settings, followed by multigene panels or exome sequencing for unresolved cases to capture *OGFRL1* and *PLCG2* variants [13,14]. Embedding molecular diagnostics within oral–maxillofacial pathways, supported by standardized referral to genetic counselling, would address the diagnostic uncertainty and limited genotype–phenotype correlation observed in the reviewed cases, enable earlier confirmation, improve surveillance planning, and support informed family risk assessment.

Developing dental genomics “expert centres” or centres of excellence has been proven to improve the management of patients with rare diseases [36]. Modelled on the European Reference Networks (ERN), these hubs for specialised diagnostics, data consolidation, training, and precision management strengthen multidisciplinary craniofacial teams and enhance patients’ experiences and support earlier case recognition. Integrating general dentists, dental specialists, paediatricians, geneticists, genetic counsellors, occupational therapists, speech pathologists, and psychosocial support will transition care from inconsistent, episodic management toward coordinated, evidence-based treatment pathways. By combining molecular insight with strengthened clinical infrastructure, Africa can enhance diagnostic accuracy, expand access to modern therapeutic options, and ultimately contribute unique scientific insights to the global understanding of rare diseases, including cherubism.

## 6. Conclusions

Cherubism remains a rare but clinically recognizable disorder, with its genetic basis increasingly well-defined. In Africa, most cases continue to be diagnosed on clinical and radiological grounds alone, with only two confirmed molecular diagnosis reported to date. Within the limitations of a narrative descriptive synthesis based on case reports and case series, these findings point to a persistent gap between phenotypic clinical recognition and genomic confirmation. Improving access to genetic testing, multidisciplinary management, and prospective registries across the continent is essential not only for improving patient care but also for ensuring that African data inform global understanding of this condition. An African-focused lens is therefore both timely and necessary, positioning the region to contribute unique insights into the genetic and clinical heterogeneity of cherubism.

## Figures and Tables

**Table 1:** Summary of Study Characteristics.

Author	Year	Country	Genetic Testing Conducted	No of. Cases	Age of Presentation (Years)	Age of Onset	Sex	Affected Jaw
Vaillant et al. [17]	1989	Algeria	NR	3	Patient 1: 13.5; Patient 2: 10.5; Patient 3: 15.5	Patient 1 + 2: 4 years; Patient 3: 3 years	1 Male; 2 Females	Patient 1: mandible; Patient 2: mandible + maxilla; Patient 3: mandible
Ayoub & Mofti [20]	1994	Egypt	NR	1	18	Shortly after birth	Female	Mandible + maxilla
Elfahsi et al. [29]	2007	Morocco	NR	1	10	4 years	Male	1st left side of the mandible then right side of the Maxilla at age of 7
Khalifa & Ibrahim [22]	2007	Egypt	NR	1	16	9 years	Male	Multiple mandibular and maxillary swellings
Temtamy et al. [23]	2012	Egypt	NR	1	14	2 years	Female	Mandible + maxilla
Elshafey et al. [21]	2014	Egypt	Yes *	1	5	9 months	Male	Mandible + maxilla
Aloni et al. [24]	2015	DRC	NR	1	10	6 years	Male	Mandible + maxilla
Idemudia et al. [27]	2015	Nigeria	NR	1	7	4 years	Male	Mandible + maxilla
Al-Omar et al. [18]	2015	Egypt	NR	1	20	Started during childhood	Female	Mandible + maxilla
Msomi & Dlamini [5]	2017	South Africa	NR	1	7	2 years	Female	Mandible + maxilla
Adam et al. [26]	2016	Sudan	NR	1	8	2 years	Male	Mandible + maxilla
Awad & Tawfik [19]	2022	Egypt	NR	5	Patient 1: 14; patient 2: 12; patient 3: 16; patient 4: 24; patient 5: 20	Patient 1 + 2: 4 years; patient 3: 8.5 years; patient 4: 3 years; patient 5: 9 years	3 Males; 2 Females	Patient 1 + 2 + 4: mandible + maxilla; patient 3: maxilla; patient 5: mandible.
Lahfedi et al. [28]	2022	Morocco	Yes **	1	12	Early childhood	Male	Mandible + maxilla
Angmorterh et al. [25]	2025	Ghana	NR	1	21	12 years	Female	Mandible

* Pathogenic variants reported (*SH3BP2*); ** Pathogenic variants not reported; NR: Not reported.

**Table 2:** Summary of family history, clinical findings and management.

Author	Clinical Appearance	Systemic Findings	Family History	Diagnosis	Management
Vaillant et al. [17]	All patients had bilateral swelling.	All patients had bilateral submandibular lymph nodes swelling and patient 3 had exophthalmos on the right eye	3 affected siblings	Clinical + radiographical + histopathological + biological investigation	Curettage + osteotomy
Ayoub & Mofti [20]	Bilateral swelling	Serum alkaline phosphatase was elevated to 16 u	NFH	Clinical + radiographical + histopathological + biological investigation	Curettage + surgical recontouring
Elfahsi et al. [29]	Bilateral swelling	Reduction in visual acuity	An older brother with history of a cherubism at age of 4 years old	Clinical + radiographical + histopathological.	Surgical: right partial maxillectomy + follow up, up to 18 months after surgery
Khalifa & Ibrahim [22]	Bilateral swelling	Serum calcium was elevated	Younger brother showed similar but less pronounced features	Clinical + radiographical + histopathological + biological investigation	Conservative + follow up
Temtamy et al. [23]	Bilateral swelling	Bilateral partial optic nerve atrophy and vision impairment+ bilateral proptosis+ conjunctivitis; severe vision impairment; and inability to close the eyes	NFH	Clinical + radiographical + histopathological	Surgery to relief pressure on optic nerve
Elshafey et al. [21]	Bilateral swelling	NAD	NFH	Clinical + radiographical + histopathological + genetic testing	NR
Aloni et al. [24]	Bilateral swelling	NAD	NFH	Clinical + radiographical + histopathological + biological investigation	Death by a sudden cardio-respiratory arrest probably due to obstructive sleep apnoea
Idemudia et al. [27]	Bilateral swelling	A positive history of nasal obstruction	NFH	Clinical + radiographical + histopathological	Conservative + follow up
Al-Omar et al. [18]	Bilateral swelling	NAD	NFH	Clinical + radiographical + histopathological	Surgical recontouring
Msomi & Dlamini [5]	Bilateral swelling	NAD	NFH	Clinical + radiographical + histopathological	Conservative, but the patient later had surgery for dental complications
Adam et al. [26]	Bilateral swelling	NAD	NFH	Clinical + radiographical + histopathological	Surgery
Awad & Tawfik [19]	Bilateral swelling	Patient 4: showed high alkaline phosphatase and serum phosphorus. Also tremors and seizures; patient 1 + 2: exophthalmos; patient 2: cervical and submandibular lymphadenopathy.	3 familial cases; (two siblings and their cousin).	Clinical + radiographical + histopathological + biological investigation	Follow up and minor surgical correction if needed.
Lahfedi et al. [28]	Bilateral swelling	Anaemia since the age of 18 months and thrombopathy	NFH	Clinical + radiographical + histopathological + genetic testing	A dental extraction with orthodontic surgery and implants were indicated after correction of anaemia and thrombopathy.
Angmorterh et al. [25]	Bilateral swelling	Enlarged right anterosuperior cervical lymph nodes	NFH	Clinical + radiographical	Did not proceed with further care in the hospital due to spiritual reasons.

NR: not reported; NAD: No abnormalities detected; NFH: No family history.

## Data Availability

No new data were created or analyzed in this study. All data supporting the findings of this review are derived from previously published literature, which is cited throughout the article. Data sharing is not applicable to this article.

## Supplementary Materials

The following supporting information can be downloaded at: https://www.mdpi.com/article/10.3390/children13020295/s1, Table S1: Search strategies used across databases.

## Abbreviations

The following abbreviations are used in this manuscript:

AJOL	African Journals Online
CT	Computed tomography
DRC	Democratic Republic of the Congo
DNA	Deoxyribonucleic acid
GOF	Gain of function
LOF	Loss of function
OMIM	Online Mendelian Inheritance in Man
OPG	Orthopantomogram
PRISMA	Preferred Reporting Items for Systematic Reviews and Meta-Analyses
SH3BP2	SH3 Domain Binding Protein 2
OGFRL1	Opioid Growth Factor Receptor Like 1
PLCG2	Phospholipase C Gamma 2
PLAID	Phospholipase Cγ2-associated antibody deficiency and immune dysregulation
SAMRC	South African Medical Research Council
H3Africa	Human Heredity and Health in Africa Consortium
