# Supplementary material for: Cherubism: An African-Focused Review
Source: Children (Basel). 2026 Feb 20;13(2):295. doi: 10.3390/children13020295 (PMC12940062; doi:10.3390/children13020295)
Supplement: Supplementary file 1 [file children-13-00295-s001.zip › children-4085002-supplementary.pdf]

Table S1: Search strategies used across databases

Database	Date searched	Search fields	Full search string
**PubMed**	20 September 2025	Title/Abstract, MeSH terms, Publication Type	cherubism[Title/Abstract] AND (Africa[MeSH Terms] OR Algeria OR Angola OR Benin OR Botswana OR Burkina Faso OR Burundi OR "Cabo Verde" OR Cameroon OR "Central African Republic" OR Chad OR Comoros OR "Democratic Republic of the Congo" OR Djibouti OR Egypt OR "Equatorial Guinea" OR Eritrea OR Ethiopia OR Gabon OR Gambia OR Ghana OR Guinea OR "Guinea-Bissau" OR "Ivory Coast" OR Kenya OR Lesotho OR Liberia OR Libya OR Madagascar OR Malawi OR Mali OR Mauritania OR Mauritius OR Morocco OR Mozambique OR Namibia OR Niger OR Nigeria OR "Republic of the Congo" OR Rwanda OR "Sahrawi Arab Democratic Republic" OR "São Tomé and Príncipe" OR Senegal OR Seychelles OR "Sierra Leone" OR Somalia OR "South Africa" OR "South Sudan" OR Sudan OR Eswatini OR Tanzania OR Togo OR Tunisia OR Uganda OR Zambia OR Zimbabwe) AND ("case reports"[Publication Type] OR "case report"[Title/Abstract])
**Scopus**	19 October 2025	TITLE-ABS-KEY	TITLE-ABS-KEY(cherubism) AND TITLE-ABS-KEY(Africa OR Algeria OR Egypt OR Nigeria OR Morocco OR "South Africa" OR "Democratic Republic of the Congo" OR DRC OR Tunisia OR Ghana OR Ethiopia OR Kenya OR Sudan OR Cameroon)
**Google Scholar**	20 October 2025	All fields (relevance-ranked)	cherubism AND (Africa OR Algeria OR Egypt OR Nigeria OR Morocco OR "South Africa" OR "Democratic Republic of the Congo" OR Tunisia OR Ghana OR Ethiopia OR Kenya OR Sudan OR Cameroon)
**African Journals Online (AJOL)**	20 October 2025	All fields	cherubism AND (Africa OR Algeria OR Egypt OR Nigeria OR Morocco OR "South Africa" OR "Democratic Republic of the Congo" OR Tunisia OR Ghana OR Ethiopia OR Kenya OR Sudan OR Cameroon)
